# Electrical, Optical and Thermal Properties of Ge-Si-Sn-O Thin Films

**DOI:** 10.3390/ma17133318

**Published:** 2024-07-04

**Authors:** Femina Vadakepurathu, Mukti Rana

**Affiliations:** Division of Physics, Engineering, Mathematics and Computer Science, and Optical Science Center for Applied Research, Delaware State University, Dover, DE 19901, USA; fvadakepurathu19@students.desu.edu

**Keywords:** infrared sensor, microbolometer, optical constants, 1/*f*-noise, thermal conductivity, 3ω method, material characterization, Ge-Si-Sn-O, germanium silicon

## Abstract

This work evaluates the electrical, optical and thermal properties of Sn-doped Ge_x_Si_1-x_O_y_ thin films for use as microbolometer sensing materials. The films were prepared using a combination of a radio frequency (RF) magnetron and direct current (DC) sputtering using a Kurt J Leskar Proline PVD-75 series sputtering machine. Thin films were deposited in an O_2_+Ar environment at a chamber pressure of 4 mTorr. The thicknesses of the thin films were varied between 300 nm–1.2 µm by varying the deposition time. The morphology and microstructure of thin films were investigated by atomic force microscope (AFM) imaging and X-ray diffraction (XRD), while the atomic composition was determined using the energy dispersive spectroscopy (EDS) function of a scanning electron microscope. The thin film with an atomic composition of Ge_0.45_Si_0.05_Sn_0.15_O_0.35_ was found to be amorphous. We used the Arrhenius relationship to determine the activation energy as well as temperature coefficient of resistance of the thin films, which were found to be 0.2529 eV and −3.26%/K, respectively. The noise voltage power spectral density (PSD) of the film was analyzed using a Primarius—9812DX noise analyzer using frequencies ranging from 2 Hz to 10 kHz. The noise voltage PSD of the film was found to be 1.76 × 10^−11^ V^2^/Hz and 2.78 × 10^−14^ V^2^/Hz at 2 Hz and 1KHz frequencies, respectively. The optical constants were determined using the ellipsometry reflection data of samples using an RC2 and infrared (IR) VASE Mark-II ellipsometer from J A Woollam. Absorption, transmission and reflection data for a wavelength range of 900 nm–5000 nm were also determined. We also determined the optical constant values such as the real and imaginary parts of refractive index (*n* and *k,* respectively) and real and imaginary part of permittivity ($\varepsilon_{1}$ and $\varepsilon_{2,}$ respectively) for wavelength ranges between 193 nm to 35 µm. An optical band gap of 1.03 eV was determined from absorption data and using Tauc’s equation. In addition, the thermal conductivity of the film was analyzed using a Linseis thin film analyzer employing the 3ω method. The thermal conductivity of a 780 nm thick film was found to be 0.38 Wm^−1^K^−1^ at 300 K. From the data, the Ge-Si-Sn-O alloy was found to be a promising material for use as a sensing material for microbolometers.

## 1. Introduction

Infrared (IR) detectors play a significant role in a wide range of applications such as military use, surveillance, medical diagnostics, remote sensing, space exploration, nondestructive inspections and scientific research involving spectrometers and radiometers. Since the 1950s, a variety of materials and technologies have been developed for IR detection. Extrinsic photoconductive detectors based on Ge:Hg and later with Au, Cu and Zn impurities in Ge were introduced in devices using long wave IR (LWIR) and very long wave IR (VLWIR) ranging from 8–30 µm spectrum [1]. Detectors made of Hg-Cd-Te were widely used for applications in short wave IR (SWIR) range but exhibit high dark current densities when operating at room temperature [2]. Detectors made of InGaAs are used in SWIR imaging, providing quality images at room temperature [3]. All these detectors require that they are cooled in order to operate. The cooling scheme adds extra costs and weight to the detector.

IR detectors can be categorized as photon and thermal detectors. In photon detectors, electron hole pairs are produced when the incident photon has more energy than the binding energy of the electrons. The spectral response of a photon detector is proportional to the wavelength. Photon detectors tend to be noisy because of the thermal generation of charge carriers, particularly at lower wavelengths. To suppress the thermal generation of carriers, these detectors require cooling which increases the complexity of the focal plane array (FPA) in the detector assembly. Thermal detectors, on the other hand, exhibit a flat spectral response across the wavelengths. Their response is proportional to thermal energy absorbed from the incident radiation. The output signal from a thermal detector depends on the radiant power or the rate of change in the incident radiation.

A microbolometer is a class of thermal detectors in which a change in temperature induces a change in the electrical resistance of the device because of absorbed radiation. Absorption of IR radiation increases the temperature of the sensing layer of a microbolometer and increases the number of carriers. The increase in carrier concentration decreases the electrical resistance of the detector which could be sensed through the read out of the electronic circuitry. The following are the figures of merits of a microbolometer.

The temperature coefficient of resistance (TCR) is expressed by Equation (1) and is defined as the percentage change in resistance, *R*, per unit change in temperature, *T*.

(1) $$TCR=\frac{1}{R}\frac{dR}{dT}$$

Also, the TCR and activation energy, *E_a_*, is related by Equation (2) at a particular temperature *T.* Since the resistance of semiconducting materials has a lower value at higher temperatures, the TCR value is negative and expressed in %/K.

(2) $$TCR=-\frac{E_{a}}{k_{B}T^{2}}$$

Here, *k_B_* is the Boltzmann constant. A higher value of *E_a_* will yield a higher value of TCR.

The responsivity of a microbolometer is a measure of the dependence of the signal output of a detector upon the input radiant power. The detector output signal may be current or voltage. Thus, the voltage responsivity, *R_v_*, given by Equation (3) is defined as the detector output voltage per unit of detector input power.

(3) $$R_{v}=\frac{\eta \alpha RI_{b}}{G_{th}(1+\omega^{2}\tau_{th}^{2}{)}^{1/2}}$$

Here,

*η* is the optical absorption efficiency of the detector;

α is the TCR;

*I_b_* is the bias current;

*G_th_* is the thermal conductivity;

ω is the modular frequency of the incident IR radiation; and *τ_th_* is the thermal time constant of the bolometer and is defined by Equation (4)

(4) $$\tau_{th}=\frac{G_{th}}{C_{th}}$$

where *C_th_* is the thermal capacitance of the device.

Voltage responsivity is expressed in V/W while current responsivity is expressed in A/W.

Detectivity, D^*^, is the area normalized signal-to-noise ratio. It has a unit of cmHz^1/2^/Watt. The Detectivity can be expressed by Equation (5)

(5) $$D^{*}=\frac{R_{v}\sqrt{A_{d}\Delta f}}{\Delta v_{n}}$$

where $\Delta v_{n}$ is the total noise voltage observed in the electrical bandwidth Δf for a detector which has an area of *A_d_*.

The Noise Equivalent Power (NEP) is the rms incident power necessary to produce a signal-to-noise ratio of unity. The unit of NEP is in Watts. NEP is related to the detectivity by Equation (6)

(6) $$NEP=\frac{(A_{d}\Delta f{)}^{1/2}}{D^{*}}$$

As seen in Equations (1)–(6), the sensing material plays the most important role for microbolometer performance. Hence, it is very important that the microbolometer material exhibits a high TCR, moderate resistivity and high absorption in the wavelength of interest and low noise.

SWIR corresponding to the wavelength range of 1–3 µm is particularly useful in starlight imaging. Optical transmission in this region is high in a wide range of materials and hence it has applications in a broader and advanced spectrum of fields like optical communication, medical diagnostics and astronomy. By virtue of the inherent low background noise involved in SWIR spectral range, they are highly valued in IR sensor development. In addition, the need for reduced computational power consumption has raised the prospects of SWIR-based sensors with advanced on-chip functionalities [4].

Si-Ge compound-based microbolometers exhibit higher TCR and hence higher detector performance. Also, the bolometer resistance can be modified by controlled doping of the material. The material property, like resistivity, depends on the scattering of carriers and number of carriers which are affected by the film growth process, among others. However, bolometers made of semiconductor films exhibit higher noise. When it comes to signal conditioning and readout circuitry for imager array development, the high resistance bolometers present difficulties in optimizing the bias currents and impedance matching with preamplifiers. Hence, a preferred option is to appropriately dope the Ge-Si compound with other materials (like C, N and Sn) to vary the resistivity and suit the applications [5].

The use of amorphous Ge_x_Si_1-x_O_y_ thin films grown by reactive sputtering with high TCR (−4.2%/K) and moderate resistivity as well as low thermal conductivity has been reported by Iborra et al. [6]. They describe the effect of deposition parameters such as substrate biasing, chamber pressure and the O_2_ content on the TCR and resistivity of Ge_x_Si_1-x_O_y_ thin films.

The use of a-Si and silicon derivatives like Ge-Si, Ge-Si-O, etc., has been reviewed by Voshell et al. [7]. The use of Ge_x_Si_1-x_O_y_ films for uncooled microbolometer was also reported by Rana and Butler [8]. Electrical, optical and noise characteristics of a 200 nm thick Ge_x_Si_1-x_O_y_ film were reported for use in microbolometers. An optimum composition of Ge_0.85_Si_0.15_O_0.035_ was reported with a TCR of −5.1%/K. They reported that the addition of Si to Ge increased the TCR from ~−1%/K to −3%/K while the addition of O_2_ in Ge-Si further increased the TCR to −5.1%/K at room temperature. Ge_0.85_Si_0.15_O_0.035_ has high resistivity, relatively higher 1/*f*-noise as well as moderate absorption in the IR region of interest.

Various atomic compositions of vanadium oxide, often known as VO_x_, have been used as one of the most popular sensing layers of microbolometers. For commercial applications, VO_x_ is the most used sensing layer for microbolometers [9]. Companies that use VO_x_ as the sensing layer for microbolometers across the world include FLIR, L-3, BAE, DRS, Raytheon, ULIS, Mitsubishi, NEC and SCD. VO_x_ thin films exhibit a TCR between −2.0%/K to −5.5%/K at room temperature depending on the stoichiometry and preparation of these films. Usually, higher TCR is associated with higher resistivity and higher 1/*f*-noise [10]. A 1/*f*-noise of ~2 × 10^−14^ V^2^/Hz at a 10 Hz frequency for tungsten-doped amorphous vanadium oxide thin films at a bias current 5 µA was also reported by Celik and Duman [11].

For uncooled IR detection, vanadium dioxide, VO_2_, is used more than any other atomic composition of V_x_O_y_ as its temperature and slope in the transition to another phase, and the resistance falloff is usually lower. Moreover, the dynamic range for bolometers made of VO_x_ is limited because of the semiconductor to metal transition at an elevated temperature of 68 °C.

Amorphous silicon, also known as α-Si or a-Si, is the second most commercially used microbolometer material after VO_x_. The most important advantage of using Si as a sensing material for microbolometers is that it is a conventional semiconductor. Being a conventional semiconductor, its processing is compatible with current complementary metal oxide semiconductor (CMOS) processing technologies. Two of the leading companies in the microbolometer industry—L-3 Communications and SAFRADIR—use α-Si as the sensing material for microbolometers. Pressure-enhanced (PE) or low-pressure (LP) chemical vapor deposited (CVD) hydrogenated α-Si (α-Si:H) is typically used as a microbolometer sensing material. The relatively lower electronic performance of low-temperature α-Si devices (mainly because of excessively high 1/*f*-noise) could be compensated by the cheaper and roll-to-roll processing technique for ultra-low-cost and high-volume applications [9]. Sylliaos et al. [12] found a TCR of −2.8%/K for the boron-doped a-Si:H and the corresponding activation energy was about 0.22 eV. They also reported a TCR of −3.9%/K for undoped a-Si:H thin films.

Low TCR and low resistivity in metal films account for the low responsivity of metals to IR radiation. High TCR together with low thermal conductance and higher absorption in the wavelength of interest play a significant role in improving the performance of the microbolometer. Our previous work reported that the addition of Sn in Ge_x_Si_1-x_O_y_ films increased the absorption in the desired IR region for microbolometer applications [13]. In this context, a Sn-doped Ge_x_Si_1-x_O_y_ film was investigated for desired electrical and optical properties for use as a NIR (SWIR) and mid IR sensor. This work investigates the electrical, optical and thermal properties such as variations of resistance with temperature, TCR, activation energy, electrical noise, thermal conductivity and variations of transmittance, reflectance and absorption as well as optical constants (n, k,${\;\varepsilon }_{1}$ and $\varepsilon_{2}$) with wavelengths of the Ge-Si-Sn-O alloy for use in microbolometers.

## 2. Materials and Methods

### 2.1. Ge-Si-Sn-O Thin Film Deposition

The Sn-doped Ge_x_Si_1-x_O_y_ films were deposited using a Kurt J Lesker, Proline PVD-75 (Jefferson Hills, PA, USA) series sputtering machine. We prepared Sn-doped Ge_x_Si_1-x_O_y_ films to obtain a TCR value >|3.5%/K| with moderate resistivity and low noise which gave us the best values for microbolometer applications. The sputtering system used two targets, each of which are 4 inches in diameter. We used a radio frequency (RF) sputtering gun for the Ge_0.85_Si_0.15_ compound and direct current (DC) sputtering gun for Sn to sputter them simultaneously at a chamber pressure of 4 mTorr in an Ar+O_2_ environment for the deposition. The sputtering power of target guns were varied to manipulate the relative concentration of constituent elements in the sputtered compound. The Ge_0.85_Si_0.15_ and Sn targets were diagonally positioned in the chamber and the substrate was rotated during the deposition to ensure uniform coating of the material. The Ge_0.85_Si_0.15_ target was prepared by bonding pieces of Si wafer using silver paste on a Ge sputtering target. The area occupied by the pieces of Si wafer determined the atomic percent of Si in Ge_0.85_Si_0.15_ target.

Ge-Si-Sn-O thin films were deposited on 0.150 mm thick and 38.1 mm^2^ cover glass substrates and Si substrates of 50.8 mm in diameter. In order to perform the thermal analysis, the thin films were also deposited on the thin film analyzer (TFA) microchips purchased from Linseis (Selb, Germany) (www.linseis.com (accessed on 27 June 2024)). The TFA microchips have a pre-defined area of a few square mm with a pre-calibrated fragile thin film of SiN on top of which the sample films were deposited. Prior to sputtering, the sputtering chamber was evacuated to ~1 to 2 × 10^−7^ Torr base pressure.

### 2.2. Thickness Measurement

The thickness of the films was measured using a Bruker Dektak-XT profilometer. For this purpose, a step profile was created using a metal dye while depositing the thin films. Measurements were repeated at three different sites on the same sample to test for measurement repeatability and to evaluate the uniformity of the sputter deposition. The film thickness was also verified by the ellipsometer model fitting during the analysis of the optical constants from the ellipsometer data.

### 2.3. Atomic Composition Analysis

The Ge-Si-Sn-O sample composition was analyzed using the energy dispersive spectroscopy (EDS) function of a Quanta FEG-250 (Hillsboro, OR, USA) scanning electron microscope. An excitation voltage of 7 keV was applied in the bulk analysis of the compound to determine the atomic weight percentage of the constituent elements.

### 2.4. X-ray Diffraction (XRD) and Surface Morphology of Thin Films

The crystal structure of the Ge-Si-Sn-O films was determined by using a Rigaku Ultima IV XRD (Tokyo, Japan) machine which uses a Cu kα1 source with *λ* = 1.5406 Å at 40 kV voltage and 44 mA current. The specimens were scanned from 2θ angles of 2° to 120°. We used Bragg–Brentano geometry with a step size of 0.020° and step duration of 1°/min. The XRD machine was equipped with a simple scintillating detector. The roughness and surface morphology of the thin films were measured using Bruker Innova, an atomic force microscope (AFM) from Bruker. The AFM used a Bruker model RTESPA-300 (Santa Barbara, CA, USA) tip in tapping mode to scan a sample with a 400.0 µm^2^ area, scan rate of 20.0 µm/s, samples/line of 512 and total lines of 512 in backward line direction using height sensor as the data type. The AFM tip had a width of 40 µm, thickness of 3.4 µm, length of 125 µm, resonance frequency of 300 kHz and force constant of 40 N/m.

We used NanoScope Analysis (Version 1.50), a software package developed by Bruker for the analysis of nanoscale surface images and data obtained from the Bruker AFM.

### 2.5. Thermal Conductivity Measurement (3ω Method)

TFA from Linseis Inc.(Selb, Germany). was used to measure the thermal conductivity of the film. We used a computer-controlled measurement setup which was equipped with a temperature-controlled (ranging from −175 °C to 280 °C) sample stage, vacuum pump, lock-in amplifier and a computer for data recording and instrument operation. The setup uses a chip manufactured by Linseis Inc. This chip has a suspended membrane setup based on the Völklein geometry for in-plane thermal conductivity measurements.

The thermal conductivity of the film was determined using Fourier’s law [14] and is expressed by Equation (7):

(7) $$\lambda_{film}=P\frac{t_{film}}{2\;w\;l\;\Delta T},$$

where *P* is the power loss and *w* and *l* are the width and length of the heater. The thermal conductivity of thin films was determined based on the *3ω* method. It uses a thin metallic strip that is in thermal contact with the sample and acts as both a heater and temperature sensor. Since the sample’s geometry is considerably bigger, the option of advanced dual membrane correction [15] can also be applied for the heat loss correction due to radiation.

### 2.6. Determination of Transmission, Reflection, Absorption and Optical Bandgap

For the optical measurements, we used a setup consisting of a blackbody light source, optical chopper, monochromator, pyroelectric detector, lock-in amplifier and a computer. By using a silicon carbide light source and monochromator (MCS 260 from Newport (Irvine, CA, USA)), different wavelengths of light were passed through the sample. This enabled the testing of the transmittance and reflection through the samples. First, the measurement was made for light passing through air. This became the total amount of light, from which the transmittance and reflectance were determined as a percentage of that total light. We used thin films deposited on cover glass substrate for this purpose.

From the transmittance and reflectance data, the absorption coefficient (*α*) of the film was determined. The absorption coefficient is the value that describes how different wavelengths of light pass through a material before it is absorbed.

Tauc’s relation for semiconductors relates the optical band gap and absorption coefficient α and can be expressed by Equation (8)

(8) $${\left(\alpha h\nu \right)}^{\frac{1}{n}}=B(h\nu -E_{g})$$

Here, *ν* is the frequency, *B* is a constant, $E_{g}$ is the energy band gap and *n* can be either ½ or 2 depending on whether the material is a direct or indirect band gap semiconductor.

The absorption coefficient *α* can be determined from the transmittance (*T*) and reflectance (*r*) data for a film thickness of *x*. This is expressed by Equation (9).

(9) $$T=\frac{{\left(1-r\right)}^{2}e^{-\alpha x}}{1-r^{2}e^{-2\alpha x}}$$

The optical band gap was determined by drawing a tangent in the linear region of the *(hαν)^2^* versus the photon energy *(hν)* graph and extrapolating it to the x-axis.

### 2.7. Determination of Optical Constants (Reflective Ellipsometry)

We used a combination of model RC2 and IR Vase Mark II ellipsometers from J.A. Woollam for the optical characterization of the thin films. The reflection data of an input polarized beam, collected for varying wavelengths within the UV-VIS-IR region and for varying angles of incidence, are matched with a fitting model to infer the optical constants—*n* and *k*. The measured values are expressed as psi (*ψ*) and delta (Δ). These values are related by Equation (10), to the ratio of Fresnel reflection coefficients $R_{p}$ and $R_{s}$ for p- and s-polarized light, respectively, and defined as complex reflectance ratio, $\tilde{\rho }$*,* of a system.

(10) $$\tilde{\rho }=\frac{R_{p}}{R_{s}}=tan\psi .e^{i\Delta }$$

After measuring the polarization states from the ellipsometry data of *ψ* and Δ, we built a multiple layer model of the sample using WVASE32 (Version 3.942), the fitting software from J.A. Woollam. WVASE32 utilizes the Snell’s law, Fresnel equations and thin-film interference based on the estimated values of *n* and *k* of the composite layer to model the *ψ* and Δ. This generated model is iteratively compared with the experimentally measured values of *ψ* or Δ and the iteration continued until the general oscillating model for the composite layer converged to the values of *n* and *k*, minimizing the mean square error (MSE).

Optical constants of real materials are defined by the complex dielectric function or complex refractive index given by Equations (11) and (12), respectively.

(11) $$\mathrm{The}\;\mathrm{complex}\;\mathrm{dielectric}\;\mathrm{function},\;\tilde{\varepsilon }=\varepsilon_{1}+{i\varepsilon }_{2}$$

(12) $$\mathrm{and}\;\mathrm{the}\;\mathrm{complex}\;\mathrm{refractive}\;\mathrm{index}\;\;{\tilde{n}}^{2}={\left(n+ik\right)}^{2}$$

${Here,\;\varepsilon }_{1}$ implies the length of propagation of the electromagnetic wave in a material after which the phase shift is 2π and $\varepsilon_{2}$ implies the power absorbed per unit time and unit volume at a point in the material.

The complex dielectric function and refractive index are related by Equation (13)

(13) $$\tilde{\varepsilon }={\tilde{n}}^{2}\;\mathrm{or}\;\tilde{n}=\sqrt{\tilde{\varepsilon }}$$

We used WVASE32 software to fit the measured data to estimate the values of the sample parameters like complex refractive index, complex permittivity of the material and thickness of the film. The Levenberg–Marquardt algorithm was employed to achieve the best fit of the model to the actual experimental data. For the UV and NIR regions (193 nm–2500 nm), measured by the RC2 ellipsometer, the model consisted of a 1 mm thick Si substrate, 723.55 nm Ge-Si-Sn-O film topped by a surface roughness layer of 8.3 nm, while for the IR region (2500 nm–35,000 nm), it was measured by the IR VASE MARK II ellipsometer, a model consisting of a 1 mm thick Si substrate, 721.27 nm Ge-Si-Sn-O film topped by a surface roughness layer of 10.97 nm. Table 1 shows the values of these parameters. The MSE value was found to be 2.276%.

The refractive index *n* of pure Ge, Si, SiO_2_ and SnO_2_ is tabulated for different wavelength ranges in Table 2. 

The RC2 ellipsometer has a wavelength range of 200 nm to 2500 nm, whereas the IR Vase Mark II has a range of operation from 1.5 µm to 30 µm. Thus, combining the data acquired from these two ellipsometers, we obtained the full range of measurements. The UV-visible spectral range is sensitive to the electronic states and excitons, whereas the IR spectral range is sensitive to the characteristic vibrations within the material. Thus, this complete range of spectrums provided valuable insights into the material properties like molecular vibrations, free-charge-carrier and phonon (lattice) absorptions involved in this compound material [21].

The data were collected at room temperature (23 °C ± 1 °C; dry ambience) and at a resolution of 8 cm^−1^. During the measurement, the number of Fourier transform infrared (FTIR) spectrometer mirror scans/spectrums (at each polarizer position) was chosen to be 15. The sample was fabricated on a Si wafer with backside roughened to eliminate the possibilities of backside reflection. Ellipsometer data for the thin film were collected at two different incident angles—55° and 75°.

The data for the Ge-Si-Sn-O thin film with a thickness of 723 nm was studied. The variation of *ψ,* Δ, *n, k* and *α* for a wavelength range between 193 nm and 35 µm is reported in this work. The layered model of the film on substrate is presented for the complete spectral range as two different models.

We used 12 Gaussian oscillators in the general oscillator model to fit the *n* and *k* or *ε_1_* and *ε_2_* across the wavelength spectrum. The model parameters including the Gaussian oscillators are listed in Table 2 and Table 3.

### 2.8. Resistivity Measurement

To measure the resistivity and noise of thin films, we cut pieces of thin film (6.35 mm^2^) deposited on glass wafer and bonded one of them on top of the cerquad chip package purchased from spectrum semiconductor (https://www.spectrum-semi.com (accessed on 27 June 2024)) using silver paste. The device was manually bonded using 150 µm thick aluminum wires with indium as the sticky agent. The spacing between the contacts where the bonding was achieved were measured using the microscope image of the specimen sample in the package.

The resistivity (*ρ*) of the thin film was calculated using Equation (14).

(14) $$\rho =\frac{R*A}{L}$$

where, *R* is the measured resistance and *A* is the cross-sectional area of the film and *L* is the separation between the probes. The resistance of the thin film was measured alongside the noise analysis of the sample which is discussed later.

### 2.9. Activation Energy and TCR

The Ge-Si-Sn-O film deposited on cover glass substrate was subjected to different temperatures (between 12 °C and 40 °C) on top of the metallic chuck of a probe station. We used a temperature-controlled probe station from Micromanipulator Inc. (Carson City, NV, USA) to vary the temperature and measured the resistance at different temperatures using a DC biasing circuit. From the variation of device resistance with temperature, an Arrhenius plot of *ln(R)* vs. *1/kT* was developed and the slope of the curve was used to calculate the activation energy (*E_a_*) of the film using the relationship mentioned in Equation (15)

(15) $$R\left(T\right)=R_{0}e^{-\frac{E_{a}}{K_{B}T}}$$

where, *R(T)* is the resistance at temperature *T* and *R_o_* is the pre-exponential factor.

### 2.10. Noise Characterization

A 9812DX low-noise measuring system from Primarius Inc. (San Jose, CA, USA) was used to measure the noise of the film at 1 µA bias current. The 9812DX system consists of (a) a Primarius 9812DX noise analyzer for detection, amplification and analysis of the low-frequency noise voltage or current generated by the device under test (DUT), (b) source measurement unit (SMU) of an Agilent model B1500A semiconductor parameter analyzer for the generation of DC biases for the DUT, as well as for measurement of *I-V* characteristics, (c) a Windows-based computer to control all the equipment through a pre-installed general purpose interface bus (GPIB) card and (d) Noisepro Plus (Version 2020.1)—a software to set up and control all the equipment to perform automatic noise and *I-V* data acquisition and analysis. For noise measurement, the sample was placed in a shielding box configured for the 9812DX system, which provided electromagnetic shielding from external interfering noise sources. The bandwidth of the high-precision current local noise amplifier (LNA) used in the measurement setup was 10 kHz. The frequency range of the noise analysis was thus limited to 10 kHz.

## 3. Results

### 3.1. Atomic Composition and Surface Morphology

**Figure 1:**
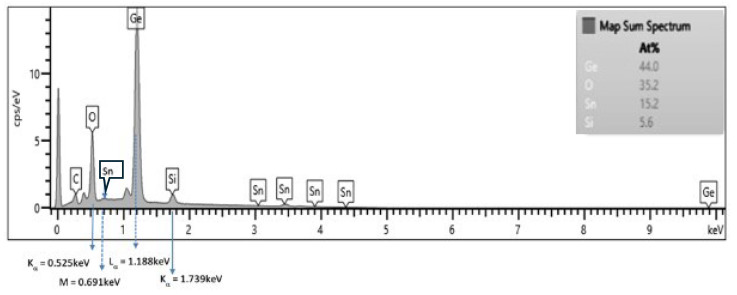
EDS (Energy Dispersive X-ray Spectroscopy) spectrum of the Ge-Si-Sn-O thin films.

The EDS analysis from the thin film is shown in Figure 1. This figure demonstrates the atomic composition of the Ge_0.45_Si_0.05_Sn_0.15_O_0.35_ thin film. 

**Figure 2:**
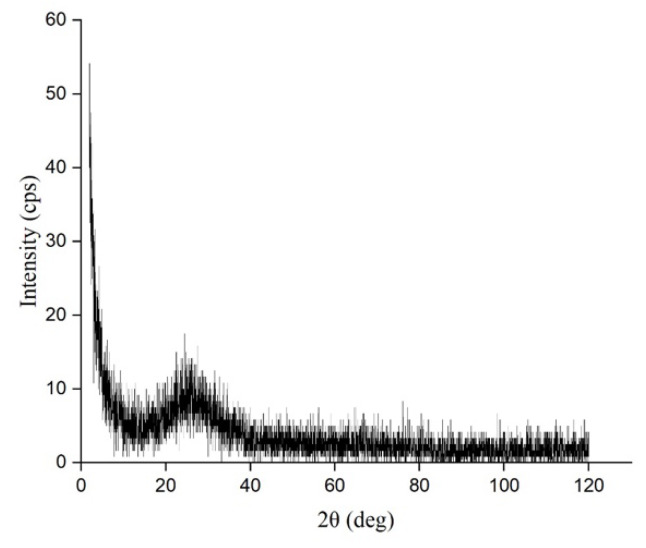
XRD pattern for Ge-Si-Sn-O film.

The XRD pattern showed (Figure 2) no sharp peaks which indicates that the compound is amorphous in nature. The Ge_x_Si_1-x_O_y_ alloy with atomic composition Si_0.125_Ge_0.8365_O_0.039_ was also reported to be amorphous in nature [22].

**Figure 3:**
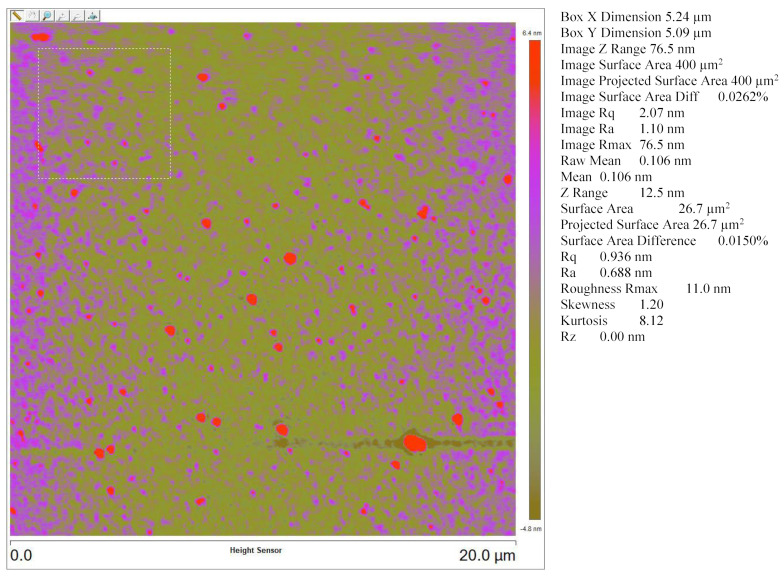
Surface profile of sputtered Ge-Si-Sn-O film determined by AFM with average surface roughness and selected area surface roughness contours (marked as a boxed on upper left corner). The right side of Figure 3 shows various parameters associated with this measurement.

The AFM image analysis demonstrated (Figure 3) an average surface roughness of 0.688 nm and root mean square surface roughness of 0.936 nm for an area of 400 µm^2^. The average roughness value is lot less than the thickness of the film and this indicates that the film thickness is uniform.

### 3.2. Thermal Conductivity

A lower value of thermal conductance is desirable as it helps to keep the temperature more stable within the material to provide a more accurate and proportional readout. A lower value of thermal conductance also blocks the heat dissipating from the sensing layer to the substrate, hence increasing the detectivity of the microbolometer. The thermal conductivity of the 780 nm thick Ge-Si-Sn-O thin film is found to be 0.38 Wm^−1^K^−1^ at 300 K. The thermal conductivity of 97 nm thick a-Si is reported as 1.5 Wm^−1^K^−1^ [23] and for 300 nm thick VO_2_ is reported as 4.2 Wm^−1^K^−1^ at 301 K [24]. Chae et al. reported the thermal conductivity of polycrystalline r-GeO_2_ to be 51 Wm^−1^K^−1^ at 300 K [25]. The thermal conductivity we obtained here is about 11 times less than VO_2_ and 3.5 times less than a-Si. A lower value of thermal conductivity will yield a higher value of responsivity and detectivity in the microbolometer.

### 3.3. Optical Properties

**Figure 4:**
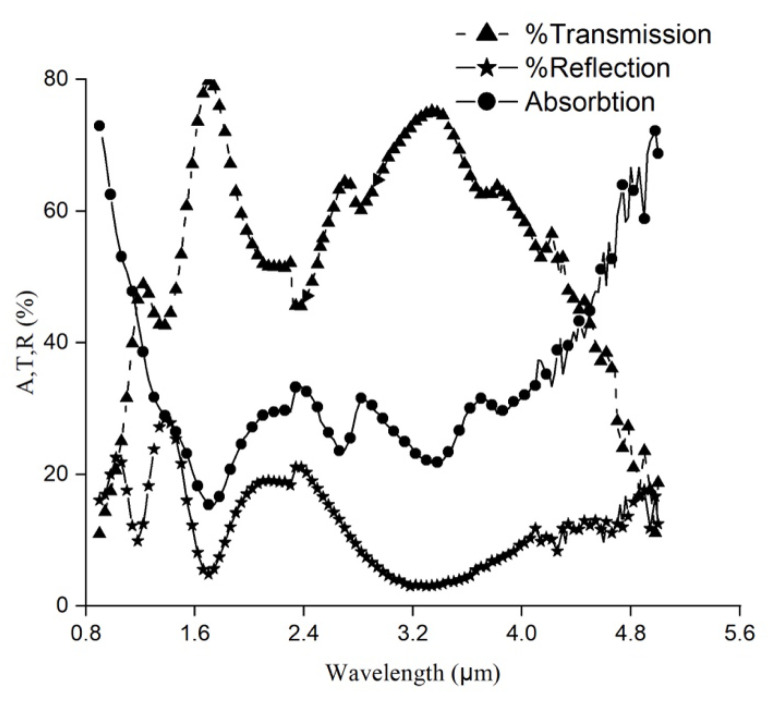
Absorption, reflection and transmission data for Ge-Si-Sn-O film.

Absorption, transmission and reflection data for Ge-Si-Sn-O for a wavelength range of 900–5000 nm are plotted in Figure 4. The grating cross-over wavelength from the monochromator configuration was 2300 nm, which accounts for the discontinuity at the same wavelength as can be seen from Figure 4.

We also observed a constant increase in the absorption of the thin film for a wavelength of >4000 nm. At 5000 nm wavelength, the absorption reached a maximum value of ~74%. The value of transmission decreased accordingly and reached a minimum value of 5000 nm wavelength. At shorter wavelengths (<900 nm), we observed the attenuation of the optical signal due to the photogeneration of carriers for photon energies greater than the optical bandgap of the material. As the wavelength increased beyond this value, the transmission through Ge-Si-Sn-O films increased as the photon energy became lower than the bandgap (1.03 eV, mentioned later). Near the 5000 nm wavelength, the absorption of the glass substrate was displayed. The higher average absorption (~30%) between 1000 nm to 4500 nm helped to increase the TCR of the thin film. Based on the variation of oxygen content between 3% and 9% in Ge-Si-O alloy, the transmission was found to vary between 60% and 90% for a wavelength range of 1500–2500 nm [26].

**Figure 5:**
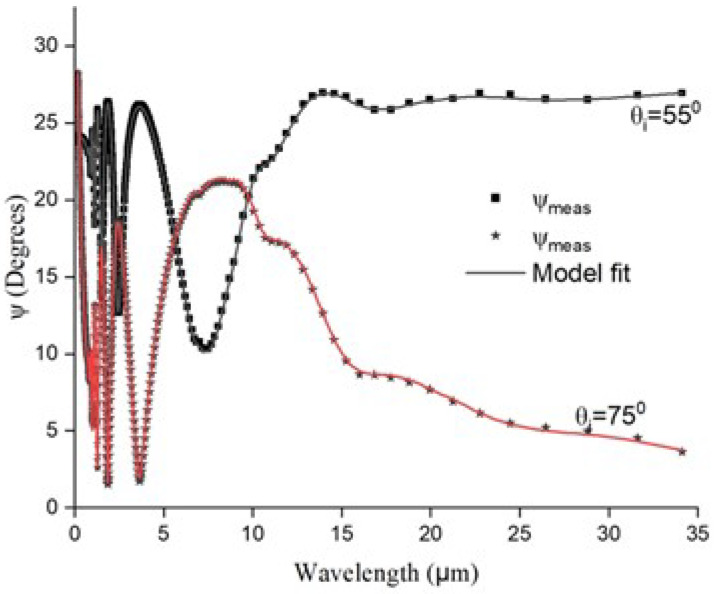
Measured ψ for Ge-Si-Sn-O film.

**Figure 6:**
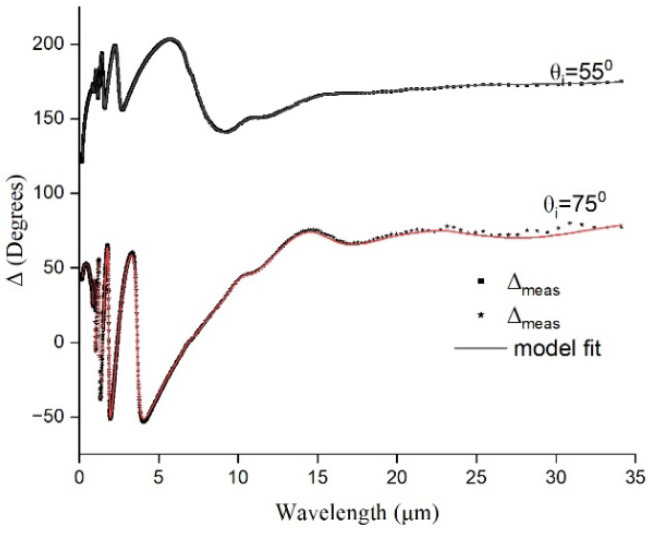
Measured Δ for Ge-Si-Sn-O film.

The measured values of *ψ* and Δ along with the model fits are plotted in Figure 5 and Figure 6. As seen from the *ψ* curves, the film is transparent for a wavelength range between 2 µm and 18 µm (NIR and portion of MIR). This is indicated by the interference peaks evident in the measured *ψ*. Beyond 18 µm, *ψ* remains less affected by the wavelength and is high for the incidence angle of 55°. *ψ* being the intensity ratio, this indicates increased absorption of IR wavelengths beyond 15 µm with an incidence angle of 55°.

The Δ curves remained around 180°, indicating that the Brewster angle is closer to 55° and *ψ* remained high above zero at this angle, indicating the increased absorption. It was also observed that the wavelength sensitivity range narrowed if the parameter of interest was Δ. Thus, material can be tuned to fit specific applications.

**Figure 7:**
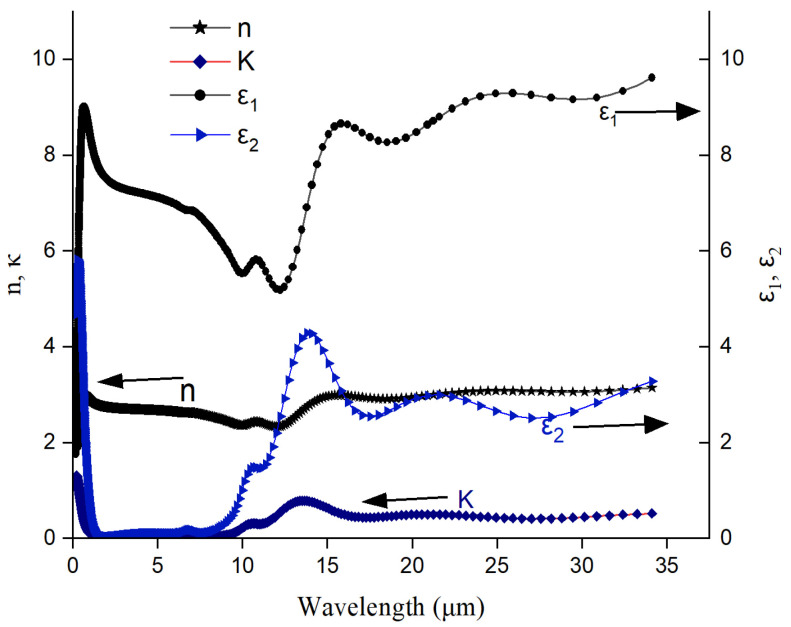
Optical constants for Ge-Si-Sn-O film.

The optical constants *n*, *k*, $\varepsilon_{1}$ and $\varepsilon_{2}$ are plotted in Figure 7, from a wavelength range of 193 nm to 35 µm for the film of Ge-Si-Sn-O.

The real part of the index of refraction (n) describes how a wave slows and bends when entering a material, while the imaginary part of the index of refraction (k) describes how a wave gets weaker as it travels. Similarly, the real part of the dielectric constant (ε_1_) describes a material’s ability to interact with an electric field (store and remit energy) without absorbing energy, while the imaginary part of the dielectric constant (ε_2_) describes a material’s ability to permanently absorb energy from a time-varying electric field.

A high value of *ε_2_* for the sample indicates higher absorption of electromagnetic energy. This is seen throughout the higher wavelengths in the MIR range. This observation is also substantiated by a positive value of *k* through the complete range of the analytical spectrum and noticeably beyond a wavelength of 6 µm. Typical absorptions can be deduced from the *ε_1_*, *ε_2_* vs. wavelengths data. It can be seen that the value of *n* is wavelength sensitive up to a range of 20 µm. A closer look into the *k* and *ε_2_* plots show a peak around 13 µm for these two parameters. This may be important for some applications specific to this wavelength. The major absorptions in the IR region can be deduced by the deconvolution of *n* and *k* graphs for identifying wavelength specific characteristics such as the chemical compositions of the alloy.

The semiconducting state optical constants for VO_2_ are reported be 2.67 and 0.04 for *n* and *k*, respectively [27]. The optical property of a V_2_O_5_ film grown by pulsed RF magnetron sputtering technique as reported in Esther et al. [28] is a thickness-dependent optical band gap of 2.59–2.78 eV. They also reported that the *n* value decreases from 1.96 to 1.65 along the solar spectral range in wavelength from 200 nm to 2100 nm. Hai et al. [29] reported the average n and *k* values for Si_0.024_Ge_0.734_O_0.242_ to be 3.87 and 0.027, respectively, over the wavelength range of 8–14 µm. The *n* and *k* values for different atomic percentage of Si an O_2_ varying between 2% and 20% in a SiGeO alloy are reported by Hai et al. [29]. The *n* value for a-Si obtained by ion implantation ranging between 3.8 and 4.0 depending on the probing wavelength is reported by Fredrickson et al. [30]. A wide range of *n* and *k* values calculated from experimentally determined dielectric constants are reported for a-Si by Aspenes and Studna [31]. This work highlights the best value of ñ = (4.086-i0.031) at a probing wavelength of 546 nm as reported by Taft [32].

**Figure 8:**
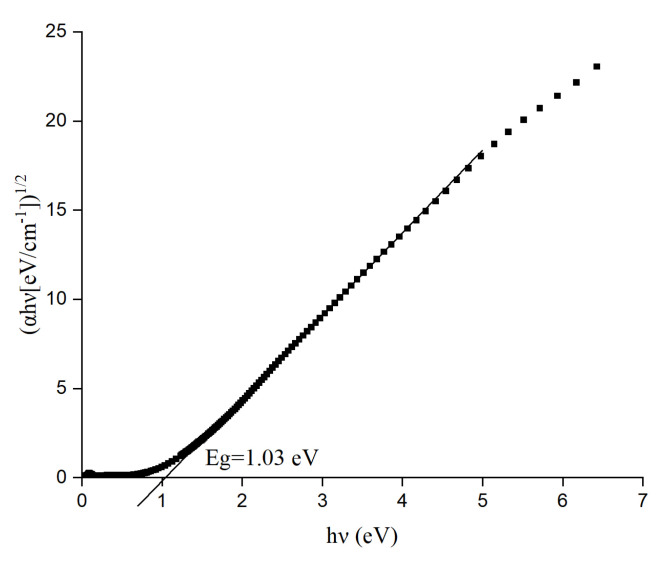
Optical bandgap for Ge-Si-Sn-O film.

**Figure 9:**
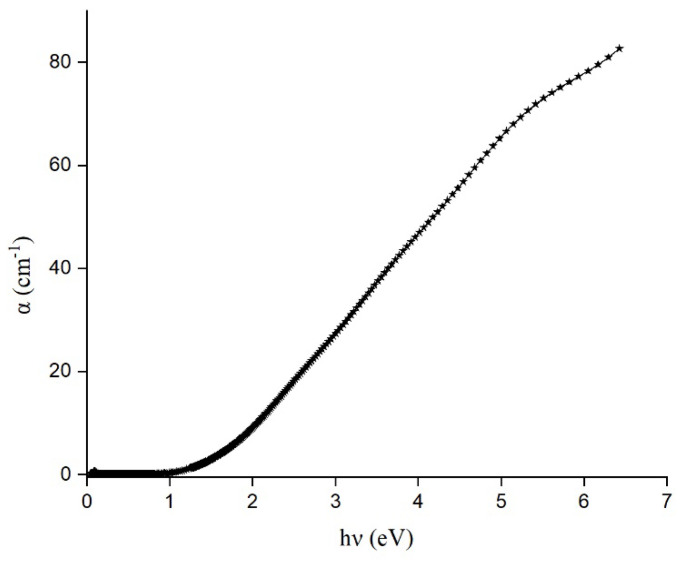
Variations in the absorption coefficient with energy for Ge-Si-Sn-O film.

The optical bandgap of the thin film was found to be 1.03 eV as shown in Figure 8. To determine the optical bandgap, we used an n value of 2 and obtained the straight line which was extrapolated in the x-axis as shown in Figure 9. The n value used here (2) to obtain the straight line associated with the graph suggests that the electronic transition is of an indirect type [33]. Rana et al. reported an optical bandgap of 0.9 eV for their RF sputtered Si_x_Ge_1-x_O_y_ thin film [26].

The variation in the absorption coefficient with photon energy determined from the reflection ellipsometry is plotted in Figure 9. From this figure, the major absorption happens in the UV-VIS region which corresponds to electronic transitions. From the Tauc equation, transparency in NIR and absorption in the UV-VIS range sheds light into the semiconductor nature of the material [21]. Once the photon energy exceeds the optical bandgap of the material (1.03 eV), the value of the *α* keeps increasing for the rest of the wavelength ranges reported here.

### 3.4. E_a_ and TCR of Samples

**Figure 10:**
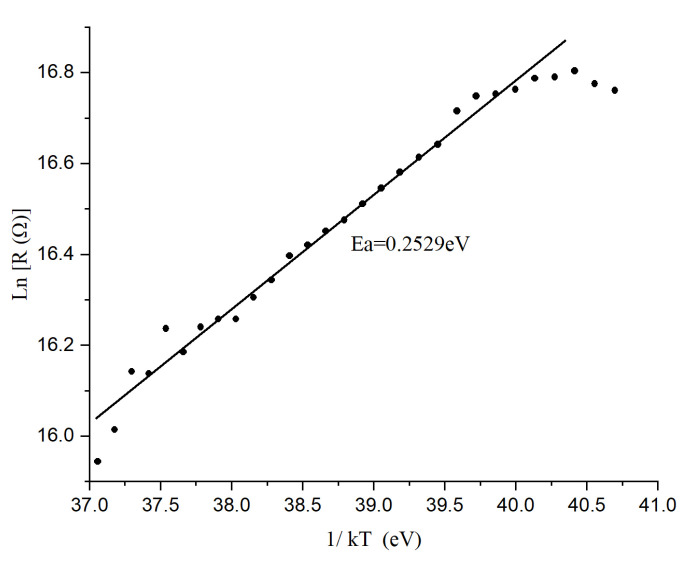
Arrhenius plot for Ge-Si-Sn-O film.

Figure 10 shows the Arrhenius plot for Ge-Si-Sn-O. The activation energy extracted from the slope of the fitting line in the Arrhenius plot is 0.2529 eV. The decrease in resistance value with increase in temperature as can be seen from the 1/kT versus Ln(R) plot proves the semiconducting behavior of the film. The data points located between the temperature ranging from 294 K to 302 K have the same slope as corresponds to the fitting line (linear relationship), indicating the thermally activated conductivity associated within that range. From the XRD data we found that the material is amorphous, and for these materials an absence of a long range of symmetry as well as the presence of defects would give rise to the presence of localized states that form the well-known extended states of the conduction and valence bands. According to Mott [34], these localized states are so-called ‘‘band tails’’ and are formed due to the disorder. There may exist another set of states in the mid-gap due to point defects in the structure. Near room temperature (294 K to 302 K), the dominant conduction mechanism is believed to be the excitation of carriers from the mid-band states into the localized band tails and hopping within the band tail states. The activation energy of conduction due to electrons excited from mid-band states (0.2529) is less than half of the optical band gap (1.03 eV).

For other temperature ranges (below and above of the range 294 K to 302 K), the data deviate from a linear relationship, indicating departure from thermally activated conductivity. This indicates the existence of other types of conduction mechanisms than the thermally activated one and needs further investigation. From this activation energy, the room temperature TCR was calculated using Equation (2) is −3.26%/K.

**Figure 11:**
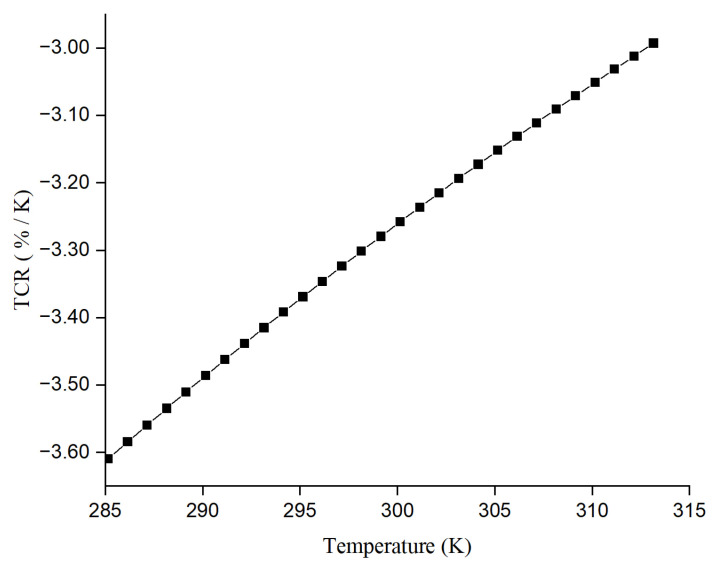
Calculated TCR vs. temp for Ge-Si-Sn-O film.

The calculated TCR versus temperature for this sample is plotted in Figure 11.

Because of the absence of work relating to the Ge-Si-Sn-O compound, we could not compare the TCR and other values with our current work. We found the closest match with the compounds of different atomic compositions from Ge-Si-O. Hai et al. [29] reported to have obtained a TCR of −4.64%/K for Si_0.080_Ge_0.856_O_0.064_ thin films of 9.48 × 10^3^ Ω-cm resistivity. Rana and Butler reported a room temperature TCR of −4.4%/K for the thin films of Si_0_._099_Ge_0_._871_O_0_._031_ [22]. The resistivity value we obtained from our thin film Ge_0_._36_Si_0_._05_Sn_0_._24_O_0_._39_ is 1.464 × 10^−3^ Ohm-cm. The resistivity and TCR values of 763 Ohm-cm and −3.518%/K, respectively, are reported for Si_0_._053_ Ge_0_._875_O_0_._072_ thin films at room temperature by Jalal et al. [35]. They reported to have TCR values ranging between −2.5%/K and −5%/K with a corresponding resistivity range from 200 Ω-cm to 1 × 10^5^ Ω-cm. VO_2_ exhibits a TCR value of around −1.86%/K subject to a post-deposition annealing at 500 °C [24]. In our case, the low resistivity value was yielded from low activation energy and hence lower TCR than Hai et al. [29] and Rana and Butler [36].

Although not presented here, in this current work we noticed that as we increase the atomic percentage of Sn in Ge-Si-O, the resistivity of the thin film decreases and so does the activation energy and TCR. In our earlier work, it was mentioned that introducing Sn in the Ge-Si-O increased the absorption [13]. The increase in absorption increased the temperature of the film which decreased resistance and hence the TCR.

### 3.5. Current Voltage Characteristics and Electrical Noise

**Figure 12:**
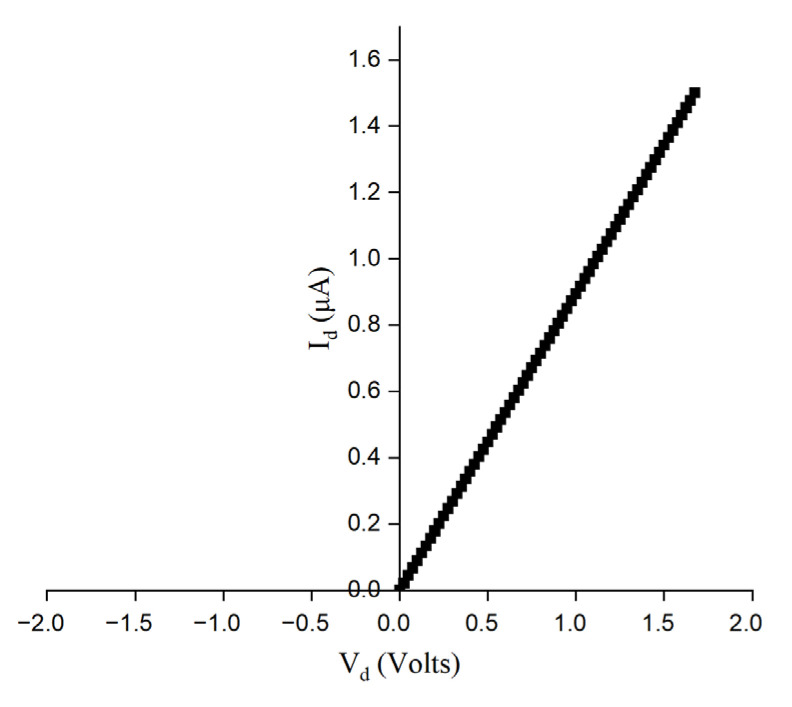
*I-V* Characteristics for Ge-Si-Sn-O film.

The *I-V* characteristics of the Ge-Si-Sn-O thin film (indium bonded on cerquad chip) is plotted in Figure 12. From the slope of the *I-V* graph, the resistance of the device is calculated to be 1.1742 MΩ. The device exhibits linear characteristics for a driving current of 1 µA. The linear resistance is measured between contact points which are 265 µm apart for a film of thickness 575 nm as estimated from ellipsometry analysis.

**Figure 13:**
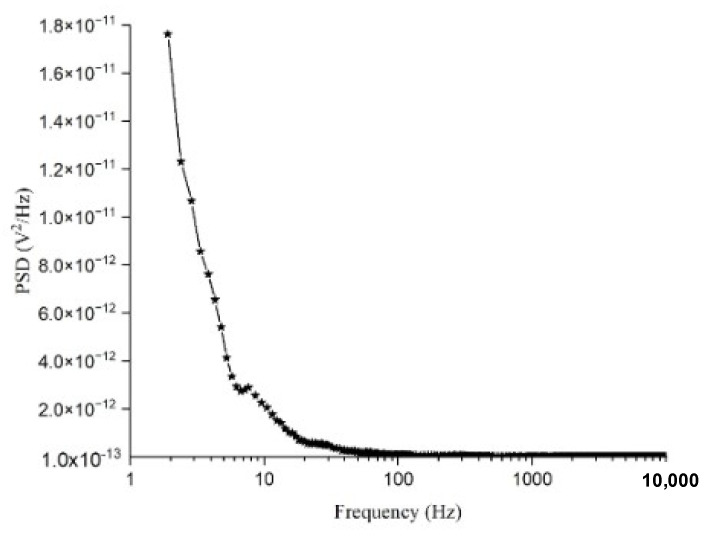
Noise voltage PSD for Ge-Si-Sn-O film.

The noise voltage power spectral density (PSD) for the Ge-Si-Sn-O film is shown in Figure 13.

The effect of noise on two of the figures of merit such as detectivity and NEP can be seen in Equations (5) and (6). The total noise from the sensing layer of the microbolometer is the sum of all the noises at a particular frequency. The lower the value of total noise or v_n_, the higher the value of detectivity. Among various noise sources, 1/*f*-noise and Johnson noise are the two major noise sources of a microbolometer. The most dominant source of noise in bolometers is 1/*f*-noise [37].

The 1/*f*-noise is dominant at lower frequencies (<100 Hz) and its value decreases by 1/10 per decade of frequency. The origin of 1/*f*-noise is still a controversial issue. Among other factors, 1/*f*-noise is dependent on the geometry, property and deposition condition of the material [38].

We can see from Figure 13 that the noise voltage PSD value decreases from 1.76 × 10^−11^ V^2^/Hz at 2 Hz to 2.28 × 10^−12^ V^2^/Hz at 10 Hz, which corresponds to almost a decade of reduction and justifies that the noise is of a 1/*f*-type. The noise voltage PSD keeps decreasing slightly to a value of 9.84 × 10^−14^ V^2^/Hz as the frequency increases from 10 to 100 Hz. In this region, the slope of the noise voltage PSD is more than a decade.

Johnson noise is caused by the random thermal motion of charge carriers in a resistive element and is not affected by the direct current flow in the circuit. The local random thermal motion of carriers sets up fluctuating charge gradients even though charge neutrality exists generally across a resistor [38]. Mathematically, this is given by Equation (16)

(16) $$V_{j}=\sqrt{4k_{B}TR\Delta f}$$

where *R* is the resistance of the device, *T* is the temperature of the detector and Δ*f* is the frequency bandwidth over which the noise is measured. As can be seen, every resistor with a finite resistance value shows Johnson noise above an absolute temperature. Johnson noise is visible at the higher frequencies and has a flat shape in the frequency spectrum.

The flat portion of the noise voltage PSD represents the thermal noise of the device which exists between 1 kHz to 10 kHz. The noise voltage PSD for this frequency range remains constant at ~2 × 10^−10^ V^2^/Hz. We calculated the resistance of the device from the thermal noise which turns out to be same as what we obtained from the *I-V* characteristics (1.17 MΩ).

Because of the absence of noise data from Ge-Si-Sn-O compounds, we used the closest and most widely used thin films’ noise data to compare for microbolometer applications. Rana and Butler reported a noise voltage PSD of 2 × 10^−9^ V^2^/Hz at a frequency of 10 Hz for their Si_0_._11758_Ge_0_._87_O_0_._0125_ thin films while the bias current was 0.3 μA [37]. For atomic compositions of Si_0_._034_Ge_0_._899_O_0_._067_ a noise value of 4.09 × 10^−13^ V^2^/Hz at 12 Hz frequency for a bias current of 0.07 μA was reported by Jalal et al. [35].

A maximum noise voltage PSD of ~1.32 × 10^−9^ V^2^/Hz at a bias current of 11.41 μA for the vanadium oxide thin films was also reported by Abdel-Rahman et al. [39].

Lopes et al. reported that for their p-type a-Si:H (boron doped) films deposited by plasma-enhanced chemical vapor deposition (PECVD) at a substrate temperature of 365 °C, the total noise was 1 × 10^−12^ V^2^/Hz at a 10 Hz frequency for a bias voltage of 3.25V and measurement at 296K [40].

Considering our 1/*f*-noise value of 2.28 × 10^−12^ V^2^/Hz at 10 Hz frequency for a bias current of 1 μA, we can conclude that this is on par as compared to other thin films which have been used for microbolometer applications.

The noise can be reduced further using forming gas passivation and silicon nitride sandwich layers as stated by Rana et al. and Jalal et al. [35,37], although further investigation is needed.

## 4. Conclusions

The electrical, thermal, optical and noise characteristics of Ge-Si-Sn-O alloy reported and discussed in this work highlights the potential of using this material as a sensing layer in uncooled IR microbolometers and could be a good competitor for a-Si and VO_x_—two of the most widely used microbolometer materials. The optical characteristics show that the material is applicable for a wide range of IR spectrum like SWIR-NIR-MIR. The effect of varying compositions of the alloy on the characteristic features could be studied further for better understanding of the material. Varying the deposition parameters as well as compositions to ensure minimization of the excess noise also could be a matter of interest.

## Figures and Tables

**Table 1:** Values of various parameters used to create models.

Parameters	For IR Vase MARK II Ellipsometer	For RC2 Ellipsometer
Film thickness (nm)	721.133	723.413
Surface roughness (nm)	10.976	8.351
Sellmeier Pole#1 Magnitude and position	217.65@15 eV	
Sellmeier Pole#2 Magnitude and position	0 @0.001 eV	

**Table 2:** Optical constant of constituent elements and their derivatives.

Material	Refractive Index n (Wavelengths)	Reference
SiO_2_	1.5 (0.2–4 µm)	Kitamura et al. [16]
SnO_2_	2.5 (0.55–0.80 µm)	Baco et al. [17]
GeO_2_	1.6 (1.7 µm)	Murphy et al. [18]
Ge	4.0 (2–14 µm)	Burnett et al. [19]
Si	3.40 (0.1–10 µm)	Wollack et al. [20]

**Table 3:** Model parameters of 12 Gaussian oscillators used for ellipsometric analysis.

Oscillator Number	Normalized Amplitude	Oscillator Energy, E_n_ (eV)	Broadening Energy, B_r_ (eV)
1	0.41899	1.5475	0.76845
2	1.8438	2.2692	1.1551
3	4.2046	7.1272	3.725
4	3.0071	3.1721	1.6249
5	4.0933	4.553	2.3695
6	3.6739	0.089599	0.023617
7	0.67692	0.11823	0.014081
8	2.7811	0.057206	0.032251
9	2.7979	0.0319	0.016921
10	0.61105	0.11171	0.042452
11	0.076722	0.18	0.016012
12	0.33629	0.055801	0.5166

## Data Availability

Data is available upon request.
